# Clean delivery practices in rural northern Ghana: a qualitative study of community and provider knowledge, attitudes, and beliefs

**DOI:** 10.1186/1471-2393-12-50

**Published:** 2012-06-15

**Authors:** Cheryl A Moyer, Raymond Akawire Aborigo, Gideon Logonia, Gideon Affah, Sarah Rominski, Philip B Adongo, John Williams, Abraham Hodgson, Cyril Engmann

**Affiliations:** 1 Global REACH, University of Michigan Medical School, 5115 Med Sci 1 1301 Catherine Street, Ann Arbor, MI 48104, USA; 2 Navrongo Health Research Centre, PO Box 114, Navrongo, UE/R, Ghana; 3 Department of Social and Behavioral Science, School of Public Health, University of Ghana, Legon, Ghana; 4 University of North Carolina, CB# 7596, 4th Floor, UNC Hospitals, Chapel Hill, NC 27599-7596, USA; 5 Department of Medical Education, University of Michigan Medical School, Towsley Center, Ann Arbor, MI 48109, USA

**Keywords:** Global health, Maternal and child health, Cord care, Developing countries, Umbilicus

## Abstract

**Background:**

Knowledge, attitudes and practices of community members and healthcare providers in rural northern Ghana regarding clean delivery are not well understood. This study explores hand washing/use of gloves during delivery, delivering on a clean surface, sterile cord cutting, appropriate cord tying, proper cord care following delivery, and infant bathing and cleanliness.

**Methods:**

In-depth interviews and focus group discussions were audiotaped, transcribed, and analyzed using NVivo 9.0.

**Results:**

253 respondents participated, including women with newborn infants, grandmothers, household and compound heads, community leaders, traditional birth attendants, and formally trained health care providers. There is widespread understanding of the need for clean delivery to reduce the risk of infection to both mothers and their babies during and shortly after delivery. Despite this understanding, the use of gloves during delivery and hand washing during and after delivery were mentioned infrequently. The need for a clean delivery surface was raised repeatedly, including explicit discussion of avoiding delivering in the dirt. Many activities to do with cord care involved non-sterile materials and practices: 1) Cord cutting was done with a variety of tools, and the most commonly used were razor blades or scissors; 2) Cord tying utilized a variety of materials, including string, rope, thread, twigs, and clamps; and 3) Cord care often involved applying traditional salves to the cord - including shea butter, ground shea nuts, local herbs, local oil, or “red earth sand.” Keeping babies and their surroundings clean was mentioned repeatedly as an important way to keep babies from falling ill.

**Conclusions:**

This study suggests a widespread understanding in rural northern Ghana of the need for clean delivery. Nonetheless, many recommended clean delivery practices are ignored. Overarching themes emerging from this study included the increasing use of facility-based delivery, the disconnect between healthcare providers and the community, and the critical role grandmothers play in ensuring clean delivery practices. Future interventions to address clean delivery and prevention of neonatal infections include educating healthcare providers about harmful traditional practices so they are specifically addressed, strengthening facilities, and incorporating influential community members such as grandmothers to ensure success.

## Background

The vast majority of the world’s four million annual neonatal deaths occur in developing countries, and more than one-quarter of those can be attributed to infections [1,2]. Although great strides have been made to reduce under five deaths with immunizations, oral rehydration and control of acute respiratory infections, neonatal mortality in developing countries remains high [3].

In Ghana, West Africa, neonatal death rates are approximately 30/1000 live births [4,5]. In northern Ghana, a vital registration system has been in place for more than 20 years in the rural Upper East region. Current estimates from the Navrongo Demographic Surveillance System (NDSS) suggest that infections are responsible for at least 20% of early neonatal deaths (newborn death within the first seven days) in the district. [6] From 1995–2002 in the same district, 23% of early neonatal deaths and 66% of late neonatal deaths (those occurring between 7 and 28 days of life) were due to infectious causes [7].

Clean delivery – including clean hands, clean delivery surface, clean cord cutting and tying, proper cord care, and bathing – is a key intervention for reducing infections in newborns [8,9]. This is especially critical in the Upper East Region of northern Ghana, where 27% of deliveries were attended by a relative or other untrained assistant, 22% of deliveries were attended by traditional birth attendants (TBAs), and only 35% were attended by nurses, midwives or physicians (the remaining deliveries were unattended or attended by community health officers) [5]. In addition, nearly half of births occur at home. [5] In such a setting, community-based understanding of clean delivery practices are critical in reducing neonatal infections.

**Figure 1:**
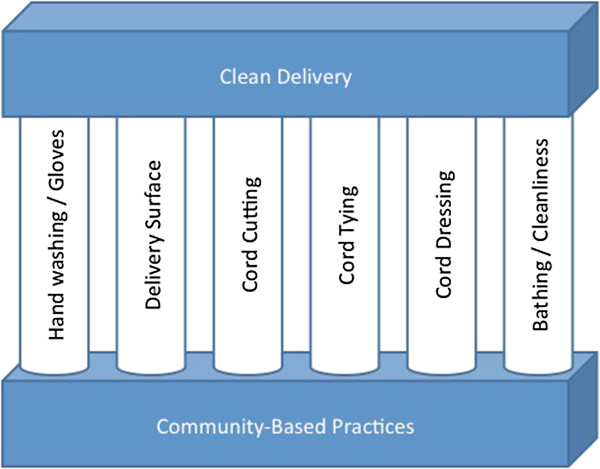
The Pillars of Clean Delivery.

Research literature to date suggests wide national and regional variability across many of these practices, especially with regard to cord care following delivery (See Table 1). With two notable exceptions [10,11], very little published data exists from sub-Sarahan Africa pursuant to community attitudes, beliefs, and behaviors surrounding clean delivery practices. Given the paucity of data and the likelihood of regional differences even within the same country, this research aimed to explore current knowledge, attitudes, and practice with regard to the six pillars of clean delivery seen in Figure 1 in the Kassena-Nankana district of the Upper East region of northern Ghana. The results of this formative study will be used to design an intervention study aimed at reducing neonatal infection. Given that over 40% of under 5 mortality occurs in the first 28 days of life [1] and the neonate has a 45 times higher likelihood of death in the first 28 days than from day 29 to 5 years, [12] we believe the results of this study can have a significant impact on not only neonatal and infant mortality in northern Ghana, but also under five mortality as well.

**Table 1:** Cord Care Following Delivery

**Substances applied to the cord**	**Country**	**Authors**
Ash	Bangladesh	Alam et al., 2008 [13]; Mullany et al., 2007 [14]
Boric Powder	Bangladesh	Moran et al., 2009 [15]; Rahman et al., 2011 [16]
Coconut Oil	Bangladesh	Alam et al., 2008 [13]; Moran et al., 2009 [15]
Cow Dung	Pakistan	Mull et al., 1990 [17]
Ghee	Pakistan	Traverso et al., 1989 [18]; Khadduri et al., 2008 [19]
Mud	Nepal	Mullany et al., 2007 [14]
Mustard Oil	Bangladesh	Alam et al., 2008 [13]; Moran et al., 2009 [15]; Rahman et al., 2011 [16]; Mullany et al., 2007 [14]; Sreeramareddy et al., 2006 [20]
Shea Butter	Ghana	Hill et al., 2010 [10]
Sunflower Seed Oil*	Egypt	Darmstadt et al., 2004 [21]
Turmeric	Bangladesh	Alam et al., 2008 [13]; Rahman et al., 2011 [16]

*As part of a randomized controlled trail, demonstrated decreased infection rates.

## Methods

### Study setting

All data were collected by the Navrongo Health Research Centre (NHRC) in the Kassena-Nankana District (KND)^1^In 2008, the Kassena-Nankana District was split into two districts – Kassena-Nankana East and Kassena-Nankana West Districts. In this study, we use the original name – Kassena-Nankana District to refer to the two districts. of the Upper East region of northern Ghana. Approximately 150,000 people live in the district, 90% in rural settlements. Subsistence agriculture is predominant, and poverty is widespread. The district has one major hospital which acts as a referral hospital to five health centres. Navrongo is the district capital, with a population of approximately 20,000. Throughout the district there are few health facilities and many transportation challenges.

### Data collection

**Figure 2:**
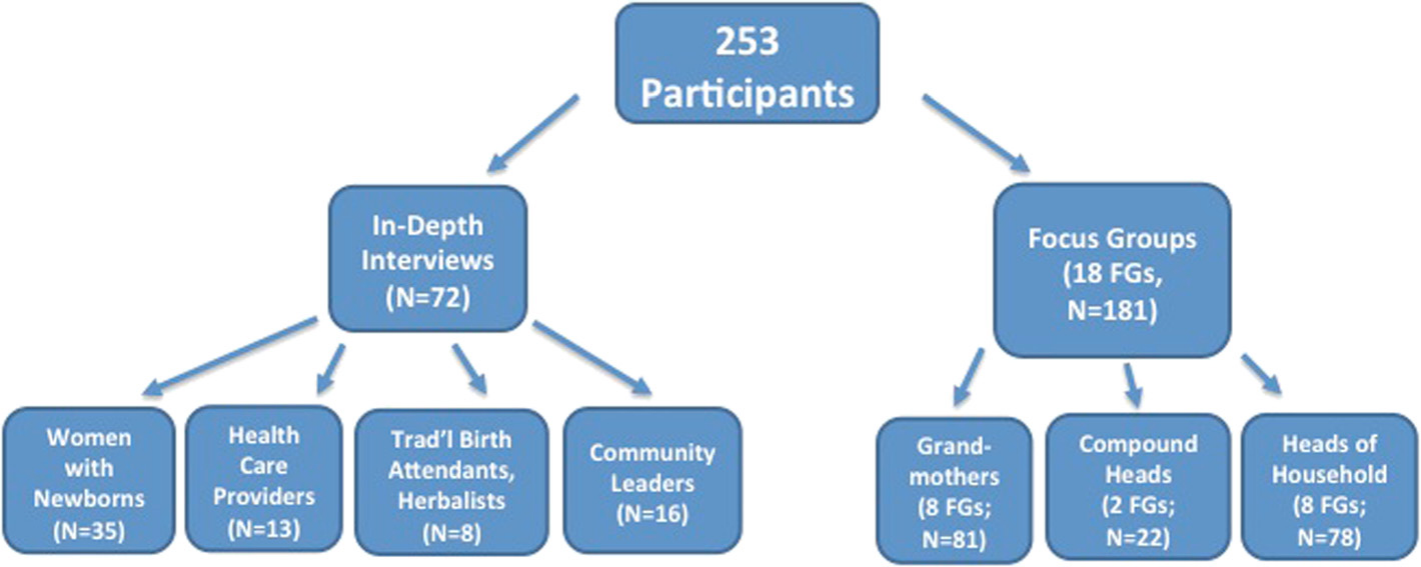
Research Participants & Operational Definitions.

In-depth Interviews (IDIs) and Focus Group Discussions (FGDs) were conducted in the KND among women with newborn infants (including those who delivered at home and those who delivered in a health care facility), grandmothers, and health care providers (both traditional and formally trained) (See Figure 2).

### Identifying participants

For NDSS data collection, the two Kassena-Nankana districts have been divided into five zones – the East, West, North, South and Central zones. The zones are further divided into clusters. Two zones were randomly selected for inclusion in this research, and within each selected zone, 12 clusters were randomly selected. Community Key Informants (CKIs) living in the communities routinely collect information on vital events including births, deaths, pregnancies and marriages, and they were contacted for a list of mothers whose infants had reached 1 month of age. The list of mothers was then categorized based on literacy, place of delivery, and number of previous deliveries. These “stratifiers” were chosen to maximize the variability of our sample, assuming that women who delivered in a facility would be likely to have different attitudes and beliefs than women who delivered at home, for example. Similarly, women who have never experienced childbirth before the recent delivery may have different perceptions than women who have had one or more previous deliveries. Within each of those groups, mothers who could be contacted immediately after the child is 29 days old were purposively selected for interview. In all, 24 interviews were planned with mothers of newborns.

Traditional Birth Attendants (TBAs), herbalists, and other local healers outside the formal health care system were purposely selected within the selected zones. Their selection was done through the CKIs who identified potential respondents based on the individual’s knowledge and/or involvement with maternal and child health at the community level.

Health care providers working in the region were also interviewed. Eight IDIs were planned with nurses/midwives. Medical assistants (the equivalent of high school graduates with less than 2 years of healthcare training) often deliver babies, thus they were invited to participate in IDIs. In Navrongo, medical doctors practice in hospitals, and there is only one hospital in the district. Thus selection of doctors for the IDIs was done at the district hospital. The Senior Medical Officer (SMO) in-charge of the district hospital was purposively selected while the second doctor was conveniently sampled on the day that the interview with the SMO was held.

With regard to focus groups, in each selected zone, five clusters were randomly selected for the purpose of focus group recruitment. CKIs who live in those communities were consulted in identifying grandmothers with relevant experience in neonatal health residing within the selected clusters. Purposively selected grandmothers were chosen to participate in an initial four focus group discussions. Each FGD included 8–10 grandmothers. Additional FGDs were added based upon the results of the first four.

Interviews and focus group discussions were conducted until thematic saturation was reached.

### The interviewers

All interviews were conducted by trained field staff employed by the Navrongo Health Research Center (NHRC). Interviewers went through at least one week-long interviewer training session lead by one of the co-investigators (RA), totaling nearly 25 h of instruction and mock interviews. All interviewers conducted a pretest interview that was reviewed and discussed to optimize data collection. Half of the interviewers had been through the training repeatedly, given that they had worked on several NHRC studies in the past.

A total of six individuals conducted the interviews and focus groups for this project. Four were Ghanaian (two were undergraduates, two were graduate students at a nearby university; three were male, one was female) and two were from the United States (both were female medical students). The American interviewers conducted interviews with English-speaking health care providers; the Ghanaian interviewers conducted all remaining interviews. Ghanaian interviewers were fluent in both the respondent’s native language (either Kasem or Nankani) but also in English, the official language in Ghana. Although the interviewers were fluent in the local languages, the interviewers did not come from the communities where the interviews were conducted. There were no known relationships between interviewers and participants.

### The interview process

In-depth interviews (IDIs) are one-on-one interviews between a field team member and a participant. Interviewers used a semi-structured instrument and detailed probes to guide the discussion. The “Saving newborn lives, tools for newborn health, 2004”, published by Save the Children [22], was used as a framework for instrument development. The interviews occurred mostly in respondents’ homes and in the health care setting (for the health care workers) and typically lasted between 45 and 60 min. Most were conducted in the mornings, but interviews were scheduled in the evenings and on weekends as necessary. All interviews were audio recorded, and notes were kept on verbal and non-verbal communication by a second field team member present at each interview. IDIs with women with newborn infants, traditional birth attendants, and herbalists were conducted in the respondent’s native language (either Kasem or Nankana). The interviews were transcribed into English, with unique words and phrases or those that were difficult to translate remaining in the local language. Interviews with health care providers were conducted in English and transcribed verbatim.

### The focus group process

Focus groups were conducted with 8–10 participants each, typically gathered in a semi-circle around the interviewer. Questions were posed to the group, and the interviewer took responses from participants one by one, moving the hand-held microphone closer to the respondent who was speaking. The note taker would keep track of respondents who appeared to be interested in speaking and would remind the interviewer to get back to that respondent before moving on to the next question. Similar to the individual interviews, FGDs typically lasted between 60 and 90 min. All focus groups were audio recorded, conducted in the local language, and transcribed into English as described above.

### Permission and invitation to participate

Permission to conduct FGDs in the community was sought from compound or community leaders. Permission to conduct the in-depth interviews at the health facilities was sought from the appropriate authorities, such as the district director of health services, the SMO in-charge of the district hospital and the medical assistants in charge of the health centers.

Information about the objectives of the discussion and the purpose of the overall study were provided to each potential participant. Confidentiality with regard to their participation and anonymity with regard to their stored data were assured, and each participant was asked for his or her verbal consent to participate in the interview or focus group discussion. Permission to audio-record the discussions was also sought and obtained.

In conducting both IDIs and FGDs, each participant was assigned a unique ID number. The numbering system reflected the type of interaction (IDI vs FGD), the type of respondent (e.g. HCP for health care provider, WNI for woman with newborn infant), the respondent’s ethnicity (Kassena vs. Nankani), and the number of the interview. Thus IDI-WNI-K2 reflected an individual interview with a woman with a newborn infant of Kassena ethnicity.

Participants did not receive any monetary incentive for participating in the discussions. However, two cakes of soap were provided as a token of appreciation for participation. This study was approved by the institutional ethics review committees of the Navrongo Health Research Center, and the Universities of Michigan and North Carolina at Chapel Hill.

### Interviewer debriefing sessions

Team debriefing sessions provided an opportunity for members of the qualitative field team to meet as a group to review and discuss their data collection activities. These debriefings were held periodically throughout the study period, and were facilitated by the project director. These sessions were designed to provide the team with an opportunity to reflect on the main themes emerging from the interviews, the degree to which interview themes or issues were showing up repeatedly, the emergence of conflicting findings as compared with information collected so far, information gaps for further follow-up, and aspects of the interview process that might need improvement. As a result of these debriefing sessions, the latter half of data collection was limited to an exclusive focus on the first seven days of life, rather than including the prenatal and extended post-natal period. In addition, these debriefing sessions led to the conduct of extra interviews with mothers and grandmothers to ensure data saturation had been reached.

### Data entry

All data were audiotaped using either digital audio-recorders or audiocassette recorders. Audiotaped data was transcribed into Microsoft Word for Windows. If portions of the audio tape were not clear, they were reviewed by all interviewers and the Project Director to determine if consensus could be reached about what was said. In the event that it was not possible to reach consensus, data were eliminated from the research record. Similarly, if there were discrepancies in interpretation through translation, all interviewers and the Project Director discussed the translation and came to consensus on the best translation. In select cases, the original word or phrase in Kasem or Nankani was left in the transcript. In addition, field notes taken using pen and paper were transcribed into the research record.

Transcripts were reviewed for obvious errors by both field staff and one of the investigators. Errors were corrected in the transcript only after discussing the transcription with the interviewer/transcriber to ensure appropriate meaning. For example, the term “outdooring” is used to denote a cultural milestone reflecting when a baby can be brought outside in Ghana. Yet when recorded and transcribed, the term “adoring” was sometimes substituted. After analysis of the context and discussion with the interviewers, such changes were made in the transcript.

### Data analysis

All interviews were read by at least three of the investigators (CE, CM, RA) and “in vivo” coding was conducted to assist in the identification of main codes. In vivo coding involves making written notes on hard copies of the transcripts and reviewing the notes together. From the in vivo coding, a preliminary coding structure was agreed upon and a codebook was created. At that point, all transcripts were entered into NVivo 9.0, a qualitative software analysis package. Focused coding (using the initial coding structure as a guide) was conducted by four separate coders, including one of the investigators (CM). The remaining three were master’s trained public health researchers.

Coders held regular coding meetings during which time the meanings of any code that came into question were revisited and discussed among the group. The codebook was revised to reflect inclusion and exclusion criteria that may not have arisen previously.

## Results

A total of 253 individuals from the Kassena-Nankana district in Ghana participated in either in-depth interviews or focus group discussions between July 1 and November 1, 2010. In-depth Interviews were conducted with 35 women with newborn infants, 13 health care providers, 8 traditional birth attendants/herbalists, and 16 community leaders. In addition, focus group discussions were conducted with 81 grandmothers, 22 compound heads, and 78 heads of household. (See Figure 2.) In this community, ‘grandmothers’ most often referred to mothers-in-law, rather than the mother’s mother. The majority of grandmothers interviewed discussed events that occurred with their son’s wife and children. All women with newborn infants, traditional birth attendants, midwives, and grandmothers were female, however the majority of community leaders, traditional healers, compound heads, and heads of household were male.

### Hand washing/glove usage

Hand washing and glove usage during delivery was rarely mentioned spontaneously by respondents.

"“We don’t use bare hands, we use gloves. And then we use clean, sterile gauze and cotton to deliver the woman. So the woman cannot get infection.” – IDI with Kassena midwife"

Health care providers expressed skepticism about the practices of the traditional birth attendants and other untrained providers with regard to hand washing:

"“Maybe the local woman was just washed her hands with water and puts the hands inside to deliver the woman…. With their dirty hands can introduce infection into the woman’s womb, can infect the child, the newborn baby,” – IDI with Kassena healthcare provider"

At least one of the traditional birth attendants described hand washing as important: “The one who is also going to assist her to deliver will wash her hand in a small plastic container and put on your gloves. … We do this because if your skin is having some dirt you can touch the baby with it and if there is any infection the baby will get it.” However, the same traditional birth attendant described using melted shea butter on her hands during pelvic examinations and delivery.

Finally, many women described needing to bring money to pay for soap for nurses to wash their hands if they delivered at a facility. “When she gets to the hospital she has to buy everything like soap so that they can use to wash hands and other things after delivery.” (IDI with women’s group leader) Table 2 illustrates the variability of responses across the groups of respondents.

**Table 2:** Representative Quotes from Respondents regarding the Pillars of Clean Delivery and Overarching Themes

**TOPIC**	**REPRESENTATIVE QUOTES**		
	**Health Care Provider**	**Traditional Birth Attendant**	**Community Member**
Hand Washing/Glove Usage	“(If) we can’t get gloves, then you wash your hands very well with soap and water. And then you do what you have to do. And the incidence of cord sepsis, septicemia, other infections, will be much, much lower.” – IDI with physician	“When you are called and you get there, you first ask if her water is broken, or better still you wash your hands and wear gloves (to) check her cervix to see if the baby has turned…” – IDI with TBA	“In the house delivery, the women who have been trained to assist in delivery do not wear anything on their hands to protect their hands and the baby. If they come they just use their bare hand like that to hold the baby. Assuming there is a cut on her hand she can infect the baby if she has any disease.” –IDI with woman with newborn infant
Delivery Surface	“Immediately the midwife removes the child, she places the child on the mother’s tummy before the cord will be cut and then all that.” – IDI with nurse	“At home birth they deliver on the floor which contains dirt and that can affect the baby with diseases, but in the clinic where you deliver on a bed it doesn’t have dirt and the floor is clean also” – IDI with TBA	“If you deliver in the filthy place the baby will get all kids of disease when the baby is still small and you will not know what to do, but when she deliver in a clean place you pick your baby very clean and bath for the baby.” FGD with Nankam grandmother
Cord Cutting and Cord Tying	“There’s a problem with the home deliveries, they may use all kinds of non-sterile things to cut the cord, or to tie the cord.” - IDI with physician	“(The cord was cut) just immediately after I delivered. It didn’t take more than 5 min.They tied it with the rag.” – IDI with TBA	“Usually they cut the umbilical cord with a blade and tie it well to stop the blood. Bath (the baby) and wrap it with rags and put it by the mother’s side on the bed for it to suck the breast milk.”- IDI with woman with newborn infant
Who Cuts the Cord?	“The cord is cut by the attending midwife or the assistant.” – IDI with physician	“The nurse will cut the umbilical cord and clean it and give it medicine.” – IDI with TBA	“It depends on those who are around to support you to deliver. If there is a nurse she will cut the cord with a scissor and if there is a TBA, she will also use a blade to cut the umbilical cord.” – IDI with woman with newborn infant
Cord Dressing	“We try to tell them to clean it. They should always clean it, make it dry… And it should be dry, it should not be wet… they should let air to it so it can dry.” – IDI with nurse/midwife	“We normally clean it and use clean Shea-butter to apply on it, is not all batter you can apply on it.” – IDI with TBA	“They use shea butter because they said if you do not use the shea butter, water will enter the sore and it will swell up.” – IDI with woman with newborn infant
Bathing/Cleanliness	“We tell them to bathe these babies twice a day, to keep the baby, the cord dry, and to wear protective clothings for the child, to sleep under ITN, and to feed this baby on demand. She should always clean her hands well when she does any work and before touching that baby.” – IDI with healthcare provider	“It is true no one wants to be dirty, how less to deliver at a dirty environment, that (is) why the hospital delivery is best. The baby is normally delivered with dirty water, and after cleaning and bathing when you place and cover the baby with dirty things, what have you done?” – IDI with TBA	“The first week if the baby is not well catered for, if it is been laid in dirty places, and if mother doesn’t bath well and clean the nipples well for the baby to suck, I think this can make the baby sick.” - IDI with assembly woman
Increasing Facility-Based Delivery	“We tell them the home, there are so many hazards. Because you can deliver and start (getting) dizzy, and then they don’t know how to arrest the hemorrhage. You may deliver the child and the child may not be breathing, or maybe if there is some placenta or cord around the, um, they will not identify it. So when they come here we identify all those things. And these days there are so many conditions, diseases.” - IDI with healthcare provider	It is better to give birth in the hospital than giving birth in the house. Because in the hospital they have everything like beds, water, drugs for delivery… Most (women) know because we have spoken to them and they understand us that the hospital have benefits for them. For that matter, they don’t call me before they go to the hospital and give birth. – IDI with TBA	“For us in the olden days we will stay in the house and will be commanding the woman to push and all of a sudden you will see the woman is lying dead and we will carry her to go and bury. So this is the reason why we have accepted the hospital for women to go and deliver there.” – FGD with Nankam grandmother
The role of Grandmothers	“Usually they don’t even allow the mother to bathe her own baby. … Because her own mother, the baby’s own mother, is not supposed to be experienced enough.” – IDI with healthcare provider	(For women who won’t breast feed) “Grandmother should see to it that they will force such women to breastfeed their children.” – IDI with TBA “It is the grandmother who is always with the baby so if the baby is not well, the grandmother will tell the mother of the baby to inform the baby’s father about it, or today I want you to take the baby to the hospital.” – IDI with TBA	“We grandmothers know how to bath the babies because their mothers don’t know, especially mothers who have delivered for the first time.” –FGD with Kassena grandmother
Disconnect between health care providers and community members	The other issue too also has to do with the perceived attitude of health workers to, to these, to these women. They (the women) feel they don’t, they, they, they get treated like equals. They don’t want to come into the hospital. Health workers are perceived to be judgmental, so a lot of people stay away from, from these hospitals.” - IDI with physician	“The baby could have ‘weni-niila’ (fit) the way the baby’s neck is turning backwards… for the ‘weni-niila’ one can go to hospital many times but western medicine cannot cure it unless the fellow uses traditional medicine…” –IDI with TBA	“Within one week you have to give a name to the baby in case the VAST worker comes around. Secondly, there are also people who come to register the babies for the birth certificates so the moment you give birth these people are around to take the name of the baby.”* – IDI with woman with newborn infant

* Traditionally, babies are not named immediately. It may take anywhere from a few days to more than a month before some families will name their infants.

### Delivery surface

Women reported giving birth on a variety of delivery surfaces, including beds with rubber sheets at the hospital, on a mattress or sheet or pile of old rags on the floor at home, in the dirt in the yard, or even on a rock.

"“I gave birth to the baby on a rock (laughing). I was attending to nature’s call (attending to toilet) when I gave birth. We were all sitting outside together when they went inside and let me alone so I decided to go towards the rock to ease myself, it was when I squatted to ease myself that the baby came out.” – IDI with Nankam mother with newborn infant"

Some women indicated that the baby stays where it was delivered until after delivery is complete: “The baby lies in the blood until the placenta comes out, or when (it) is taking too long the cord is cut and baby is bath while the cord tied,” (Women’s group leader) Other women and most health care providers report that the baby is immediately placed with its mother. “Immediately the midwife removes the child, she places the child on the mother’s tummy before the cord will be cut and then all that.” (IDI with healthcare provider (nurse))

Most women appreciated the need for a clean delivery surface as a means of preventing infection in their babies:

"“They have been telling us to deliver in the facilities because they have beds and everything to deliver a woman, but at home you will just deliver on the floor which is very dirty and can make the baby sick,” – IDI with Kassena woman with newborn infant"

"“In the local houses they can deliver a woman in the sand if it becomes critical, but for the nurses, even if it is an emergency they will clean the place before the woman will deliver to avoid the baby from get any infections. The old women, when it becomes critical, they can deliver you in the middle of the road when they do not have anything to spread on the ground for you to deliver on and they have also said such things will give the baby some infections.” – IDI with Nankam woman with newborn infant"

Healthcare providers reiterated the importance of a clean delivery surface: “Oh that is very paramount because, uh, if the environment is not clean, clearly there will be infection. Not only to the woman but also to the newborn. Yes. And clearly we have septicemia as one of the major causes of neonatal mortality here.” – IDI with healthcare provider (physician)

Healthcare providers also indicated that finding a clean delivery surface during home deliveries can be challenging, given the agrarian nature of much of the region: “I am talking about especially in the rural areas, you see the typical compound is around these, um, animal farms. But they also have animals in the yard which are being bred. The animal dung is being collected as manure during the farming season… which is very good. But the problem is, during the dry season or even the rainy season, before you enter the home you have to step on this. And then you get it into the room, more or less infecting the room every now and then. You see. And that is a problem.” – IDI with healthcare provider

Most respondents – including mothers, grandmothers, community leaders, and health care providers – indicated that clinic deliveries were more likely to yield a clean delivery than home deliveries.

### Cord cutting and cord tying

Respondents indicated that the umbilical cord was cut within a few minutes of delivery, but exactly who cut the cord varied. (See Table 2) Some respondents reported adhering to traditional practices during cord cutting, including covering the mother’s face so she could not see what was happening. “They say it will spoil the eyes of the mother that is why they use the broken calabash to cover their faces before they will cut the umbilical cord.” – IDI with Nankam mother with newborn infant

According to respondents, the cord is typically cut with scissors or a razor blade. Among the 35 recently-delivered women in our sample, 16 women reported the use of scissors, 6 reported the use of a razor, and 13 admitted they didn’t know what was used.

Unprompted, some women described the importance of sterility when cutting the cord: “People are saying that they use contaminated instruments to cut the cord thus infecting it with tetanus, but in the clinic it is not like that so that is why everyone want to deliver in the clinic.” – IDI with Kassena mother with a newborn infant

Respondents indicated a variety of non-sterile tools with which the cords were tied, including rags, twigs, a piece of linen, a piece of string, a small rope, or a plastic clamp. Respondents described the importance of tying the cord to stop bleeding as well as to prevent infection, but local health care providers expressed concerns about traditional practices. “There’s a problem with the home deliveries, they may use all kinds of nonsterile things to cut the cord, or to tie the cord.” – IDI with healthcare provider (physician)

### Cord dressing

When asked about how the cord was cared for following delivery, community members reported unanimous awareness of the need to treat the cord appropriately. As one grandmother reported,

"“When you are also treating the cord, you have to take good care of it so that dirt will not enter it for it to get rotten and breed maggots because this is where you will not feel comfortable taking care of it.” – FGD with Nankam grandmother"

"“Since there is a sore, if … the one bathing the baby does not blow out the water from the navel after bathing the baby, it will rot. That is why we use shea butter on it to cover the red part.” – FGD with Nankam grandmother"

To prevent excessive moisture and dirt from getting on the cord, mothers and grandmothers report covering the cord with shea butter, ground shea nuts, local herbs, local oil, or “red earth sand.” (See Table 3) Another community member described using the juice of a local plant to assist in cord healing.

**Table 3:** Summary of Cord Treatment in our Sample (N = 35)

**Was anything put on the umbilical cord?**	
Don’t know =	6 (17.1%)
No =	4 (11.4%)
Yes =	25 (71.4%)
**What was put on the umbilical cord?**	(27 out of 35 provided a response)
12/27 (44.4%)	Shea Butter Alone
6/27 (22.2%)	Combination (oil and spirits, spirits and plaster, earth and juice from pou plant, shea butter and ground shea nuts)
4/27 (14.8%)	Nothing
3/27 (11.1%)	Oil Alone
1/27 (3.7%)	Ground Shea Nuts Alone
1/27 (3.7%)	Spirits (alcohol)

Health care providers reported that women are advised to put nothing on the cord. One midwife said that she advises women that “if there’s no infection it necroses and then dry off.” Another midwife reiterated:

"“They shouldn’t use anything. Like this local things like cow dungs. That eh, that is the, the olden days. They used the cow dungs to cover the cords. And some use shea butter to cover the cord up. So that can bring, especially cow dung, it can bring infection. Especially tetanus. Neonatal tetanus. And now they don’t do it.” – IDI with healthcare provider (midwife)"

### Bathing/cleanliness

Cleanliness (known as “yeera Kweem” or personal hygiene in Kassem), and maintaining clean surroundings were mentioned in a variety of contexts, including breastfeeding, sleeping quarters, and bathing babies frequently.

Women, grandmothers, and community leaders spoke about the need to keep a woman’s breasts clean prior to breastfeeding an infant:

"“The nurses have taught us that immediately the baby is born and you cut the umbilical cord and the baby cries you have to teach the mother wash her nipples very well. Then you hold the breast into the baby’s mouth.” – FGD with Nankam grandmother"

They also spoke about the need for general cleanliness: “When the woman returns from nature’s call she has to wash her hands before she can pick/carry the baby. If the woman does not wash her hands and picks the baby, the baby can get some infection and become sick.” – FGD with household head

Health care providers also reported that new mothers needed to pay more vigilant attention to cleanliness than other community members:

"“They (young mothers) should be sure every morning they should clean their surroundings, wash their cooking utensils, fetch good drinking water, and then the food they eat, especially the mother if they eat it, clean, cooked food so that she doesn’t get any sickness. And then where the baby is lying should be clean. All the baby’s clothes should be clean. Then they should bathe, the mother and the baby should bathe morning and evening.” – IDI with healthcare provider (nurse)"

Respondents reported that clean sleeping quarters were an important part of preventing infections.

"“After delivery you have to make sure that where the baby is sleeping is clean of dirt so that the baby will not be infected because we know that most of the infections are through dirty places.” FGD with Nankam household head"

"“If the sleeping place is not clean or if you don’t wash it clothes or if you don’t cover it well. It can get cold and fall sick.” – IDI with Kassena woman with newborn infant"

Bathing was mentioned frequently across all types of respondents, the majority of which cited the relationship between frequent baths and reducing the risk of infection.

"“If you cannot keep good hygiene the infections will be there like that. If you do not wash the clothes of the baby and do not dry them if the baby wets them when you wake up in the morning but keep them like that it will give the baby some infections.” – FGD with Nankam grandmother"

Bathing appears to be most often left to the purview of grandmothers. To the question, “Who typically baths the babies?” the response in a focus group of grandmothers was resounding: “We, the ‘kazina’” (Kasem for grandmother) This response was reiterated among women with newborn infants. Out of the 35 women with newborn infants who were interviewed, 24 of them indicated it was their mothers or their husband’s mothers who bathed their babies.

There was general agreement that warm water was used to bathe the babies, but no consensus as to why warm water was used rather than cold water. One Nankam mother’s response was that they “use warm water because the baby’s body is not yet strong.”

Data also suggested inconsistencies with regard to when the babies were bathed. Some mentioned bathing infants shortly after delivery, while others mentioned waiting until later in the day to bathe. None described waiting more than a few hours before bathing.

### Overarching themes

Data suggested several overarching themes that supersede the six prevention practices. First, the proportion of women in the community who deliver their infants in facilities appears to be increasing. Second, grandmothers have an extremely powerful social position that influences young mother’s behavior. Finally, there appears to be a disconnect between health care providers and the community members in terms of their understanding of ideal and actual maternal and child health behaviors.

### Increasing facility based delivery

As one new mother said, “Delivery at clinic and home delivery are not the same because you can deliver safely in the clinic since they have everything there.” (IDI with Nankam mother with newborn) Data indicate that attitudes toward delivering at a facility are changing throughout the community, with grandmothers, community leaders, and compound heads all suggesting that facilities are the safest places for women to deliver. (See Table 2.)

### Grandmother’s role

Another key finding in these data relates to the role of the grandmother in this rural region in Ghana. As was described, grandmothers are frequently in charge of bathing infants. They are also frequently cited as sources of information, decision-makers, and elders who command respect and are in a position of authority.

"“It is the old ladies (that) advice them about where and how to sit to make their delivery easy and how it lies when the pregnancy is about some months.” –FGD with Nankam household head "

In several interviews with women with newborn infants, grandmothers nearby repeatedly engaged in conversation and provided answers for the new mothers, even after being asked to let the women answer themselves. The women interviewed also repeatedly said they would do what their mothers and mother-in-laws told them, because that is how it is done. It was even suggested that the grandmother may have more influence than the baby’s father: “Even though the father can also (give) advice… but that is what we the women do here.” – FGD with Kassena grandmother

### Disconnect between providers and the community

Finally, the data collected in this study suggest that health care providers and community members are not always in agreement about what is happening in the community or what is happening in the health care facilities. One example of the disconnect between providers and new mothers in the community relates to what is being put on the umbilical cord. Health care providers unilaterally say that they tell women to put nothing on the cord. Yet more than 70% of the women in our sample said they dressed the cord of their newborn with one of a variety of substances. (See Table 3) This example speaks to a broader issue of the relationship between patients and providers in this community. While many patients and providers expressed mutual respect for one another, Table 3 illustrates an example of the language that was repeatedly used to describe the tension between uneducated rural women and the nurses and midwives in the health centers. “Health workers are perceived to be judgmental, so a lot of people stay away…” – IDI with healthcare provider (physician)

## Discussion

The data presented here suggest that there is widespread understanding of the need for clean delivery practices to reduce the risk of infection to both mothers and their babies. This understanding expands beyond the health care setting, where it might be expected, and into the rural community. Despite this understanding, when looking at six key clean delivery practices that can be targeted for intervention – hand washing/use of gloves during delivery, delivering on a clean surface, sterile cord cutting, appropriate cord tying, proper cord care following delivery, and infant bathing and cleanliness – each appears to have room for improvement in this rural area in Northern Ghana. Notably, behaviors appeared to differ based upon delivery location – with facility-based deliveries and deliveries attended by skilled birth attendants much more likely to comply with clean delivery recommendations than home deliveries and those attended by traditional or untrained attendants.

In our study, the use of gloves during delivery and hand washing during and after delivery were mentioned infrequently, despite repeated discussion of the need for cleanliness to avoid infant infections. This may be due in part to the open-ended nature of the interview tool – respondents were not directly asked, “What about hand washing?” However, it is noteworthy that discussions prompted by “What needs to be clean during delivery?” rarely included discussion of hand washing. Respondents frequently mentioned the need for a clean delivery surface, including explicit discussion of avoiding delivering in the dirt. However, at least 3 of the 35 recently-delivered mothers in our sample delivered their infants on the way to the facility, including deliveries alongside the road. Cord cutting was done with a variety of tools, the most common of which were razor blades or scissors. Cord tying also utilized a variety of non-sterile materials, including string, rope, thread, twigs, and clamps. These data suggest that applying traditional salves to the cord – including shea butter, ground shea nuts, local herbs, local oil, or “red earth sand” – is still a common practice in this region of Ghana. The motivation behind doing so appears to be to prevent infection – “so that dirt will not enter it for it to get rotten.” Finally, these data suggest an appreciation for the need to bathe infants frequently and keep their surroundings clean in order to prevent infection.

Our findings also suggest that this community is undergoing a shift toward a greater percentage of facility-based deliveries. These findings mirror results published elsewhere: in the northern region where our study was conducted, 2003 data suggests that 29% of women delivered in a health facility [23], whereas 2009-2010 data suggest that number has risen to nearly 70% (data not shown). This mirrors trends seen on the national level [5]. While this is good news from the perspective of the World Health Organization’s recommendation that all deliveries be attended by a skilled provider [24], it also suggests that facilities in this region need to be equipped to handle the increasing volume and complexity of patients. This may require additional staffing, re-training of existing staff, addition or renovation of physical infrastructure and close attention to quality improvement techniques. Perhaps most importantly, the technical skills of the providers need to be supplemented with an understanding of long-held traditional practices and beliefs. Community members and providers need to work together to ensure that birth traditions can be upheld in as clean a manner as possible in order to reduce cultural barriers to facility-based delivery.

These data also suggest that grandmothers are critical social gatekeepers, providing advice, guidance, and advocacy regarding how mothers and their babies ought to behave and be treated. While such a finding has been demonstrated in the context of breastfeeding [25-28], and while research in Ghana has supported the involvement of husbands as financial decision-makers [29], to date the literature has yet to demonstrate the importance of grandmothers in preventing neonatal infection. The data presented here suggest that future interventions and health promotion efforts are likely to be more successful if grandmothers are incorporated into the program planning and implementation phases.

Finally, these data suggest that health care providers and community members are not always in agreement with regard to maternal and child health practices. This suggests that not only do healthcare providers need to be educated about broad community perceptions, but they need to proactively ask about them with individual patients. Perhaps most importantly, providers need to be willing to discuss these issues openly and respectfully and work with patients and family members to find acceptable alternatives to traditional (or standard medical) practices. Providers also need to appreciate that unless acceptable solutions are devised, agreed-upon, and implemented collectively with a woman and her extended family, traditional practices may be resumed once she leaves the hospital. The results presented here suggest that researchers and policy makers need to engage health care providers and community members in working together to help plan interventions that maximize community participation.

Our findings complement those by Hill et al., who reported on clean delivery practices in Central Ghana. [10] They interviewed women who had recently delivered (30 IDI and 2 FGD), traditional birth attendants and grandmothers (20 IDI and 6 FGD), and husbands (12 IDI and 2 FGD), and analyzed the prevalence of clean delivery behaviors collected through a demographic surveillance system. In their study, they report that most women delivered on a covered surface, and had birth attendants who washed their hands, cut the cord with a new blade and tied it with a new thread. They also described as near universal the frequent application of products to the cord. Husbands were singled out as key in financial decision-making, thus the authors suggested incorporating them in home visits [29]. There are also important contrasts to the work by Hill et al. Our findings suggest that in Northern Ghana there may be a greater use of non-sterile materials to tie the cord such as twigs, string, rope and thread, as well as the use of contaminating materials on the umbilical cord, such as red earth soil, as well as local herbs and oils. While husbands were singled out as prime drivers of newborn healthcare decision in central Ghana, we noted the prominent role grandmothers play in the north.

Our findings also supplement four other studies in Africa that have addressed prevention of neonatal infection through community-based practices [11,30-32]. All four were intervention studies, yet Meegan et al. used their knowledge of community practices to inform and shape their intervention. The authors conducted an evaluation of the effect of a health-promotion program on neonatal tetanus among the Maasai in Kenya and Tanzania. While traditional cord care among the Maasai includes packing the umbilical stump with cow dung, the authors were able to work with local leaders to encourage substituting washing the stump with water or milk instead. This resulted in a dramatic drop in neonatal tetanus rates (0.75 per 1000 births in the intervention areas vs 82 per 1000 births in the control areas). This is an example of working with the community to provide an acceptable substitute for long-held traditional infant care beliefs, something that our data suggest will be critical if we are to address neonatal infection rates in rural areas of developing countries.

Our results also complement research conducted outside Africa. For example, Sreeramareddy et al. (2006) [20] found that in Nepal, only 16.2% of mothers who delivered at home used a clean home delivery kit, only 38.3% of the birth attendants had washed their hands prior to delivery, and nearly 94% of infants were given a bath shortly after birth [20]. As seen in Table 1, our finding regarding non-sterile substances being applied to the cord is not uncommon in the developing world. Mustard oil was applied to the cord in 22.1% of deliveries in Nepal.

We believe the research reported here has several key strengths. First, this study represents 253 individual respondents who completed in-depth interviews or participated in focus group discussions in 2010. The study includes a diversity of perspectives, including women with newborn infants, grandmothers, compound heads, community leaders, formally-trained healthcare providers, and traditional healers. It also includes diversity within each of those groups. For example, among mothers, our sample includes women who delivered unassisted, assisted by a traditional birth attendant, or assisted by a skilled birth attendant. It includes women who delivered at home and in a variety of types of facilities, from the health center to the district hospital. It includes literate and illiterate women, women experiencing their first birth or one of many. And it includes women of both Nankani and Kasem ethnicity. This comprehensive approach helps ensure that our findings reflect a rich and variable portrait of newborn care in this region – including the influences of grandmothers, compound heads, community leaders, and health care providers.

Despite its strengths, there are limitations to this study. First, interviews were conducted by undergraduate- and graduate-student interviewers. It is possible that results might have been different if the community members perceived the interviewers to be more similar to themselves. It is also possible, on the other hand, that community members were less guarded among students than they might have been with local peers. Given the volume of information readily volunteered and the 20-year history of conducting interviews in the community we believe respondents were not inhibited by the student status of interviewers. Finally, the design of this study did not include an assessment of actual infections resulting from post-natal practices. Future experimental research is needed to assess the relationship between traditional and contemporary practices in rural northern Ghana in order to document actual neonatal infection rates. However, given the formative, hypothesis-generating nature of this research, we believe our findings provide useful information to researchers, clinicians, and program planners in Ghana and beyond.

The results presented here provide an important backdrop against which future interventions can be planned. Newborn-care interventions are not new – the NewHints Trial in central Ghana [10], Nepal’s Safe Delivery Incentive Programme (SDIP) [33], and the Pregnancy and Village Outreach Tibet (PAVOT) program [34] are just a few examples of programmatic attempts to improve the way infants are handled upon delivery. However, our results suggest that future interventions would benefit from thoughtful inclusion of grandmothers and other key community figures in addition to training traditional birth attendants and others who might attend home deliveries. Our results suggest that grandmothers play a very important role in infant care and must not be overlooked as important stakeholders with regard to infant care. In addition, our results uncover a notable disconnect between providers and community members – one that must be breached if future interventions are going to be successful.

## Conclusions

In summary, our research suggests that in this region in northern Ghana, clean delivery is afforded a high priority among health care providers and community members. However, despite a widespread understanding of the importance of cleanliness as a means to preventing neonatal infections, practices do not always reflect adequate implementation of clean delivery practices, especially in community settings. Grandmothers play a key role in all areas of post-delivery care, as do traditional community beliefs about what is appropriate. Health care providers appear to differ from community members in their views of ideal post-delivery care. This research raises critical questions about the disconnect between health care providers and community members and suggests that future maternal and child health interventions would be well-served to include grandmothers and other community figures.

## Endnotes

^a^In 2008, the Kassena-Nankana District was split into two districts – Kassena-Nankana East and Kassena-Nankana West Districts. In this study, we use the original name – Kassena-Nankana District to refer to the two districts.

## Competing interests

The authors declare that they have no competing interests.

## Authors’ contributions

CAM, MPH: Ms. M was involved in the conceptualization of the research, the development of the survey instrument, the development of the protocol, the coding and analysis of data, and the preparation of the manuscript. RAA, MPH: Mr. A was involved in the conceptualization of the research, the development of the survey instrument, the development of the protocol, the coding and analysis of data, and the preparation of the manuscript. GL, BA: Mr. L was involved in primary data collection as well as the coding and analysis of data and the preparation of the manuscript. GA, BA: Mr. A was involved in primary data collection as well as the coding and analysis of data and the preparation of the manuscript. SR, MPH: Ms. R was involved in the coding and analysis of data and the preparation of the manuscript. PB A, PhD: Dr. A was involved in the conceptualization of the research, the development of the survey instrument, the development of the protocol, the coding and analysis of data, and the preparation of the manuscript. JW, MD: Dr W was involved in the conceptualization of the research, analysis of data, preparation of the manuscript, and providing leadership and supervision to team members at the Navrongo Health Research Centre. AH, MD, PhD: Dr. H was involved in the conceptualization of the research, analysis of data, and preparation of the manuscript, and providing leadership and supervision to team members at the Navrongo Health Research Centre. CE, MD: Prof. E was involved in the conceptualization of the research, the development of the survey instrument, the development of the protocol, the coding and analysis of data, and the preparation of the manuscript. All authors read and approved the final manuscript.

## Footnotes

^1^In 2008, the Kassena-Nankana District was split into two districts – Kassena-Nankana East and Kassena-Nankana West Districts. In this study, we use the original name – Kassena-Nankana District to refer to the two districts.

## Pre-publication history

The pre-publication history for this paper can be accessed here:

http://www.biomedcentral.com/1471-2393/12/50/prepub
